# Mitochondrial Membrane Damage Is Prevented by an Anthocyanin-Rich Fraction of *Callistemon citrinus* in 6-OHDA-Exposed SH-SY5Y Cells

**DOI:** 10.3390/biom16081144

**Published:** 2026-08-06

**Authors:** Martina Farina, Giuseppe Tancredi Patanè, Stefano Putaggio, Ester Tellone, Davide Barreca, Alessandro Maugeri, Michele Navarra

**Affiliations:** 1 Department of Chemical, Biological, Pharmaceutical and Environmental Sciences, University of Messina, 98168 Messina, Italy; martina.farina@studenti.unime.it (M.F.); giuseppe.patane@studenti.unime.it (G.T.P.); stefano.putaggio@studenti.unime.it (S.P.); ester.tellone@unime.it (E.T.); davide.barreca@unime.it (D.B.); mnavarra@unime.it (M.N.); 2 “Prof. Antonio Imbesi” Foundation, University of Messina, 98100 Messina, Italy; 3 Institute of Biomedicine (iBiMED), Department of Medical Sciences, University of Aveiro, 3810-193 Aveiro, Portugal; 4 Department of Veterinary Sciences, University of Messina, 98168 Messina, Italy

**Keywords:** neurodegenerative diseases, Parkinson’s disease, anthocyanins, *Callistemon citrinus*, *Melaleuca citrina*, 6-OHDA, neuroprotection, SH-SY5Y, oxidative stress, natural products

## Abstract

Neurodegenerative diseases represent a significant clinical challenge. Understanding the pathogenic mechanisms is essential for developing more effective therapies, and mitochondria appear to play a key role in these processes. Since current treatments are limited to symptom management, natural strategies capable of preserving mitochondrial function could represent a promising preventive and therapeutic approach in neurodegeneration. This study aims at investigating the molecular mechanisms underlying the neuroprotective potential of an anthocyanin-rich extract from *Callistemon citrinus* flower (Cce) in differentiated SH-SY5Y cells exposed to 6-hydroxydopamine (6-OHDA). Exposure of cells to 6-OHDA inhibited cell viability and caused cell death, events hindered by the pre-treatment with Cce. Furthermore, it restored normal cell cycle distribution, as well as hampered 6-OHDA-induced apoptosis. Given the pro-oxidant effect of 6-OHDA, we observed that Cce reduced reactive oxygen species in stressed SH-SY5Y cells, along with recovering their antioxidant protection system. Focusing on mitochondria, Cce was able to protect their membranes from 6-OHDA, as suggested by the restoration of mitochondrial membrane potential. This led to a reduction in release of cytochrome c and the consequent activation of caspases 9 and 3, characteristic of the intrinsic apoptotic pathway, supporting our initial findings. Our results indicate that Cce prevents SH-SY5Y cell death induced by 6-OHDA via the preservation of mitochondrial integrity mainly through to its antioxidant properties, encouraging further studies to support its exploitation in the management of neurodegeneration.

## 1. Introduction

Neurodegenerative diseases represent one of the most demanding health challenges of this century, with a steadily growing global epidemiological impact [1]. Among these, Parkinson’s disease (PD) ranks second as the most common neurodegenerative disorder after Alzheimer’s one, and first as the most common disorder of movements in the world [2,3,4]. According to the Global Burden of Disease, approximately 11.77 million people were affected by PD globally in 2021, with age-standardised rates increasing compared to 1990, and projections indicate a further rise in the burden of this disease up to 2035 [4,5]. From a neuropathological perspective, PD is defined by the loss of dopaminergic neurons in the substantia nigra pars compacta and the presence of Lewy bodies, intracellular structures composed primarily of aggregated α-synuclein [6]. Concurrently, a broad convergence of experimental and clinical evidence indicates that mitochondrial dysfunction constitutes one of the central pathogenic mechanisms in PD, both sporadic, which account for the vast majority of cases (80–90%), and in familial forms (10–20%) [7]. In fact, dopaminergic neurons, which are characterised by exceptionally high energy demands, are particularly vulnerable to impaired mitochondrial function. This can lead to a bioenergetic deficit, the generation of reactive oxygen species (ROS), altered mitochondrial dynamics, and the induction of apoptosis due to oxidative stress. Several gene mutations have been ascribed to directly interfere with these processes, confirming the central role of mitochondrial biology in the aetiopathogenesis of PD [8]. Therefore, mitochondria represent a therapeutic target of primary interest in PD, as well as other neurodegenerative diseases [9]. Despite advances in understanding the molecular mechanisms, currently available therapies for PD remain predominantly symptomatic, lacking the ability to modify disease progression [10]. This situation necessitates the search for new therapeutic strategies in order to prevent the onset of neurodegenerative pathologies.

In this field, there is a growing scientific interest in naturally occurring active compounds as potential preventive and therapeutic agents for neurodegenerative diseases. In particular, polyphenolic compounds have been shown to modulate critical cellular pathways involved in neuroprotection, including the reduction in oxidative stress, the modulation of inflammation and the restoration of mitochondrial function [11,12]. Recent reviews have further highlighted how these molecules may represent safe and accessible sources for the development of innovative therapies for neurodegenerative diseases, acting directly on the restoration of mitochondrial biogenesis, mitochondrial fusion/fission and cellular redox balance [13,14].

*Callistemon citrinus* (Curtis) Skeels (syn. *Melaleuca citrina*), commonly known as the ‘bottlebrush’ or ‘lemon bottlebrush’, is an evergreen shrub belonging to the Myrtaceae family endowed with remarkable adaptability. Indeed, this species has become widely established as an ornamental plant throughout the world [15]. Alongside this use, *C. citrinus* has been employed for centuries in traditional medicine to treat a wide number of human pathologies [15]. Phytochemical screening of various parts of the plant, including leaves, stem bark, flowers and seeds, revealed the presence of a wide plethora of secondary plant metabolites [16,17]. The pharmacological potential of *C. citrinus* is supported by a steadily growing body of the experimental literature. The reported therapeutic properties include anti-cancer in both colon [18] and breast [19], anti-hyperglycaemic [20], antimicrobial [21], hepatoprotective, cardioprotective and gastroprotective ones [22,23]. Moreover, we recently investigated the effects of *C. citrinus* flower extract for its application in the food industry to prolong shelf life [24]. Of particular note, recent studies have highlighted an inhibitory effect on neurodegeneration [25], opening up promising prospects in the context of neurodegenerative diseases. On this line, the aim of this study was to investigate the molecular mechanisms underlying the neuroprotective potential of an anthocyanin-rich extract from *C. citrinus* flower in differentiated SH-SY5Y cells exposed to 6-hydroxydopamine (6-OHDA).

## 2. Materials and Methods

### 2.1. Preparation of Anthocyanin-Rich Fraction from C. citrinus (Cce)

Fresh blossoms of *C. citrinus* were collected in Messina (Sicily, Italy) in May–June 2024 and processed once their residual moisture content was below 2%. To obtain an anthocyanin-rich extract (Cce), the polyphenolic constituents were first extracted and then subjected to a selective enrichment step of anthocyanins, following the environmentally sustainable procedure previously reported by Patanè et al. [24]. Briefly, by means of solid-phase extraction on C18 cartridges, non-anthocyanin phenolics were largely removed from the total polyphenols initially extracted, as confirmed by HPLC analysis [16]. The resulting Cce was recovered as dry powder, with a yield of 22.3 ± 1.9%, stored at 4 °C in the dark and re-dissolved immediately before use. For both biological assays and analytical determinations, fresh stock solutions were prepared under light-protected conditions and used immediately to limit pH- and light-induced degradation of anthocyanins.

### 2.2. Qualitative and Quantitative Characterisation of Cce

The Cce was characterised by reverse-phase high-performance liquid chromatography coupled to diode array detection and electrospray ionisation tandem mass spectrometry (RP-HPLC-DAD-ESI-MS/MS), as previously reported [24]. The analyses were performed on a ThermoQuest LCQ-Duo system equipped with a DAD and an ion-trap mass analyser with an ESI source operating in positive ion mode. The separation was obtained on a Luna Omega PS C18 column. The mobile phase consisted of solvent A (water with 0.1% formic acid) and solvent B (acetonitrile), delivered in a linear gradient. The UV–Vis spectra were acquired between 200 and 550 nm, while specific wavelengths were checked to verify the presence of different polyphenolic compounds. Mass spectrometric detection was carried out using nitrogen as drying gas (9 L/min, 40 psi, 350 °C). Helium served as the collision gas for collision-induced dissociation (CID) in MS/MS mode (1.46 × 10^−5^ bar), with a fragmentation amplitude of 1.0 V. Compound identification relied on comparison of retention times and UV–Vis absorption features and compared with previously reported data [16,26]. Quantitative results were expressed as cyanidin-3-O-glucoside (CyG) equivalents per 100 g of dry extract (DE).

### 2.3. Cell Culture and Treatment

The SH-SY5Y human neuroblastoma cell line and human peripheral blood mononuclear cells (PBMCs) were originally obtained from ATCC (Rockville, MD, USA). Both were kept in RPMI 1640 medium at 37 °C in a humidified atmosphere of 5% CO_2_. Regarding SH-SY5Y cells, for each experiment described below, the initial differentiation protocol consisted in the exposure of cells to 10 µM retinoic acid (Sigma-Aldrich, Milan, Italy) for 5 days in low-serum medium, as previously reported [27,28]. For both, all reagents for cell culture were from Euroclone (Milan, Italy). The working solutions of Cce were prepared by dissolving the lyophilized powder in culture medium to obtain the working concentrations, which were immediately used. The stressor we employed was 6-OHDA (Sigma-Aldrich), which was dissolved in phosphate-buffered saline (PBS) immediately prior to use and added to cultured cells in the presence of the Cce pre-treatment or clean medium for 6-OHDA-only stressed cells, to reach a concentration of 50 µM.

### 2.4. Cytotoxicity Assays

Cell viability was evaluated by the resazurin assay, as previously reported [29]. Both differentiated SH-SY5Y cells and PBMCs were seeded into 96-well plates and treated with Cce at different concentrations (from 6.25 to 100 µg/mL) for 24 h to assess the safety profile of the extract. Having defined the safe concentrations, we treated differentiated SH-SY5Y cells with Cce (12.5, 25 and 50 µg/mL) for 1 h and added 6-OHDA 50 µM (Sigma-Aldrich) for a further 24 h. After treatments, plates were centrifuged to remove supernatants, which were substituted by fresh media containing 0.01% *w*/*v* resazurin (Santa Cruz, Dallas, TX, USA). Plates were kept at 37 °C for a further 3 h. Fluorescence was measured via a microplate reader FLUOstar Omega (BMG Labtech, Ortenberg, Germany; ex: 544 nm, em: 590 nm). The viability was expressed as the percentage of living cells in treated cultures compared to untreated ones.

Cell death was assessed by the lactate dehydrogenase (LDH) release assay (TOX7, Sigma-Aldrich), following manufacturer’s guidelines [30]. Differentiated SH-SY5Y cells were seeded and treated as previously reported for the resazurin assay. Plates were centrifuged and then 50 µL of supernatant from each well were transferred to a clean plate, where a further 50 µL of freshly prepared LDH assay mixture was added. The plates were kept in the dark for 30 min at room temperature. The reaction was ended by adding an acidic solution and absorbance was quantified spectrophotometrically at 490 nm with a microplate reader FLUOstar Omega (BMG Labtech). The LDH release was expressed compared to that of untreated cells, which was arbitrarily defined as 1.

### 2.5. Cell Cycle Analyses

The assessment of cell cycle distribution in 6-OHDA-stressed SH-SY5Y cells pre-treated with Cce was carried out as previously reported [31]. After treatments, cells were washed with ice-cold PBS. After centrifugation, cell pellets were resuspended in ice-cold 70% ethanol and kept at 4 °C overnight. Afterwards, cells were centrifuged, washed twice with ice-cold PBS and resuspended in a solution of propidium iodide (PI; 50 µg/mL) in PBS. Stained samples were immediately acquired using a Novocyte 2000 cytofluorimeter, assessing 10,000 events per sample (ACEA Biosciences Inc., San Diego, CA, USA).

### 2.6. Detection of Apoptotic Cell Death

The effect of Cce against 6-OHDA-induced apoptosis was investigated employing the Annexin V-fluorescein isothiocyanate (FITC)/PI staining (Invitrogen, Carlsbad, CA, USA) [32]. In brief, SH-SY5Y cells were seeded in 6-well plates and pre-treated for 1 h with Cce (12.5, 25 and 50 µg/mL) and 6-OHDA 50 µM (Sigma-Aldrich) was added for a further 24 h. Afterwards, cells were collected and stained first with Annexin-V for 15 min in dark, and then with PI. Stained samples were analysed employing a Novocyte 2000 cytofluorimeter, assessing 10,000 events per sample (ACEA Biosciences Inc.).

### 2.7. Real-Time PCR Analysis

For the gene expression studies, SH-SY5Y cells were seeded and treated as explained above. The total RNA was extracted using PureZol reagent (EuroClone) and reverse transcribed with High-Capacity cDNA Archive Kit (Applied Biosystems, Life Technologies, Foster City, CA, USA). Quantitative PCR (qPCR) reactions were carried out in triplicate in 20 µL containing 1× SYBR Select Master Mix (Applied Biosystems), 0.2 µM of specific primer pairs (Table 1) and 25 ng of cDNA. The analysis was performed on a QuantReady K9600 real-time PCR System (LifeReal Biotechnology Ltd., Erreci, Milan, Italy). As housekeeping control, we employed *ACTB* and a standard dissociation stage was included to evaluate primer specificity. Data were gathered and analysed using the 2^−∆∆CT^ relative quantification method. Values are expressed as fold change relative to control cells, as previously reported [29]. The primer sequences employed for real-time PCR are listed in Table 1.

### 2.8. Cytofluorimetric Determination of ROS and Mitochondrial Membrane Potential (ΔΨm)

The quantification of ROS was performed by using 2′,7′-dichlorodihydrofluorescein diacetate (DCFH-DA; Sigma-Aldrich) while modifications of mitochondrial membrane potential (ΔΨm) were estimated by quantifying rhodamine 123 (R123; Sigma-Aldrich) incorporation [33]. Briefly, cells were seeded in 6-well plates and treated with Cce prior to being exposed to 6-OHDA 50 µM for 6 h. Afterwards, cells were washed and incubated with DCF-DA (20 μM) or R123 (1 µM) in PBS for 30 min, protected from light. The fluorescence of 10,000 events per sample was acquired with a Novocyte 2000 cytofluorimeter (ACEA Biosciences Inc.).

### 2.9. Evaluation of Catalase (CAT) and Superoxide Dismutase (SOD) Activities

Catalase (CAT) and superoxide dismutase (SOD) activities were assessed by using commercial assay kits (ab83464 and ab65354, Abcam, respectively) [28]. Briefly, cells were plated in 6-well plates and, after 24 h, were pre-treated with Cce for 1 h and exposed to 6-OHDA 50 µM for 24 h. Afterwards, cells were washed with ice-cold PBS, lysed with the provided buffer, and centrifuged. The assays were carried out on supernatants according to the manufacturer’s protocols. The absorbance was recorded by a microplate reader FLUOstar Omega (BMG Labtech) at 450 nm for SOD and 570 nm for CAT.

### 2.10. Quantification of Glutathione (GSH) Content

Levels of glutathione (GSH) were quantified using a commercially available kit, following manufacturer’s procedures (MAK440, Sigma-Aldrich) [28]. After treatments, cells were detached from dishes and pellets were lysed in cold neutral EDTA buffer, with or without scavenger. After centrifugation, samples were deproteinized and centrifuged. Supernatants were put into a 96-well plate and diluted in assay buffer. Finally, 100 µL of working reagent was added to each well and fluorescence was recorded immediately and after 10 min with a microplate reader FLUOstar Omega (BMG Labtech).

### 2.11. Assessment of Malondialdehyde (MDA) Levels

For the evaluation of lipid peroxidation, we quantified the amount of malondialdehyde (MDA) in SH-SY5Y cells following the manufacturer’s guidelines (MAK568, Sigma-Aldrich) [34]. Briefly, cells were seeded in a 100 mm Petri dish and treated as above. Afterwards, cells were detached employing lysis buffer and centrifuged at 13,000× *g* for 10 min. The supernatants were mixed with an acidic solution of 2-thiobarbituric acid and tubes were incubated for 1 h at 95 °C. Samples were cooled on ice and 200 µL were aliquoted in triplicate in a clear 96-well plate to read the absorbance at 530 nm with a microplate reader FLUOstar Omega (BMG Labtech).

### 2.12. Quantification of Cytochrome c Release

The assessment of cytochrome c release was achieved employing a commercially available kit, following manufacturer’s procedures (ab221832, Abcam) [35]. Briefly, cells were grown in 6-well plates and treated as above. After detachment, pellets were lysed with extraction buffer and protein content quantified. After dilution in buffer, samples were added to the provided plate and kept for 1 h. Then, wells were washed and developer solution was added for 6 min. Finally, stop solution was added and absorbance was acquired at 450 nm with a microplate reader FLUOstar Omega (BMG Labtech).

### 2.13. Assessment of Caspases Activity

The enzymatic activity of caspase 3 and 9 was measured using a commercial kit (ab39401 and ab65607, Abcam, Cambridge, UK) [28]. Briefly, cells were grown in 100 mm Petri dishes and then treated as described above. Cells were lysed and supernatants were mixed with reagent mixtures, different for the two caspases, in equal volume in a 96-well plate. After incubation at 37 °C for 1 h, absorbance was measured at 405 nm for caspase 3 activity, while fluorescence was recorded for caspase 9 activity with an excitation wavelength of 355 nm and an emission wavelength of 460 nm by a microplate reader FLUOstar Omega (BMG Labtech).

### 2.14. Statistical Analysis

The statistical assessment for differences was performed via one-way analysis of variance (ANOVA), using Dunnett’s multiple comparison test (GraphPad Prism Software for Science, version 8.4.2, San Diego, CA, USA). Results of three independent biological replicates (*n* = 3) are expressed as mean ± standard error of the mean (SEM).

## 3. Results

### 3.1. Quali-Quantitative Composition of Cce

The Cce used in this study comes from the same batch employed by Patanè et al. [26]. On the basis of retention time, UV–Vis spectral features and MS/MS fragmentation patterns, four individual anthocyanins were identified and quantified as CyG equivalents (526.00 ± 5.95 mg CyG/100 g DE). Among these, cyanidin-3,5-O-diglucoside represented the 55.75 ± 0.48% of total CyG equivalents in DE, being 293.27 ± 2.51 mg CyG/100 g DE, while peonidin-3,5-O-diglucoside the 35.04 ± 0.46%, being 184.31 ± 2.41 mg CyG/100 g DE, being by far the major components, followed by cyanidin-3-O-glucoside (7 ± 0.06% of total CyG in DE, 36.84 ± 0.33 mg CyG/100 g DE) and a cyanidin-coumaroylglucoside-pyruvic acid derivative (2.20 ± 0.14% of total CyG in DE, 11.57 ± 0.71 mg CyG/100 g DE). No detectable signals attributable to other polyphenol subclasses were recorded at 260, 292, 330, or 370 nm wavelengths, typically employed for monitoring the non-anthocyanin phenolic compounds. Data highlight that the phenolic compounds in Cce were predominantly represented by anthocyanins. The precise quali-quantitative profile of anthocyanin present in Cce was already reported [26].

### 3.2. Safety Profile of Cce in SH-SY5Y Cells

**Figure 1:**
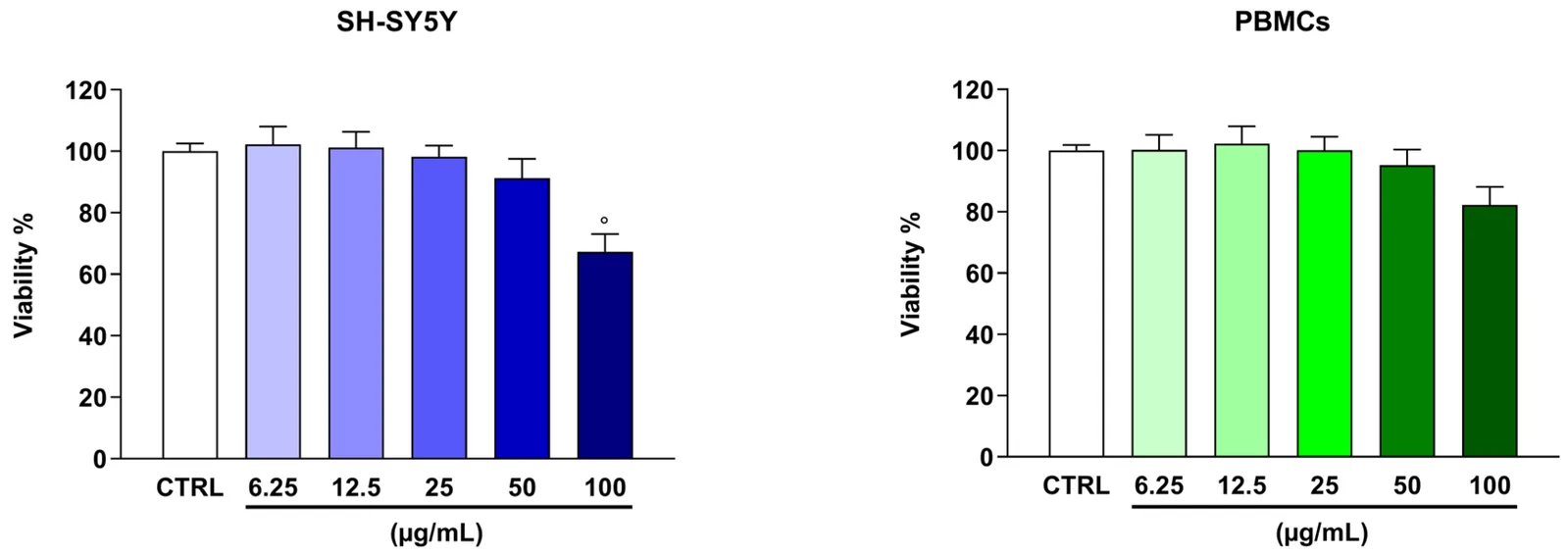
Effect of Cce on the viability of differentiated SH-SY5Y cells and PBMCs. Cells were treated with different concentrations of the extract (from 6.25 to 100 µg/mL) for 24 h. Viability was assessed by resazurin assay. Results are expressed as the percentage of fluorescence detected in cells compared with untreated ones, set as 100% (CTRL). Data are expressed as means ± SEM of three independent experiments performed in eight replicates (*n* = 3). ° *p* < 0.05 vs. CTRL.

The safety profile of the extract was assessed in differentiated SH-SY5Y cells to select the concentrations to be employed in further experiments. The treatment of cells with our extract (6.25–100 µg/mL) for 24 h did not exert any sign of toxicity, except from the highest one (Figure 1). Therefore, we excluded this concentration and proceeded with the lower ones.

Notably, Cce did not reduce the viability of normal human PBMCs, further confirming the safety of our extract (Figure 1).

### 3.3. Cce Protected Viability of SH-SY5Y Cells and Prevented Their Death from 6-OHDA Exposure

**Figure 2:**
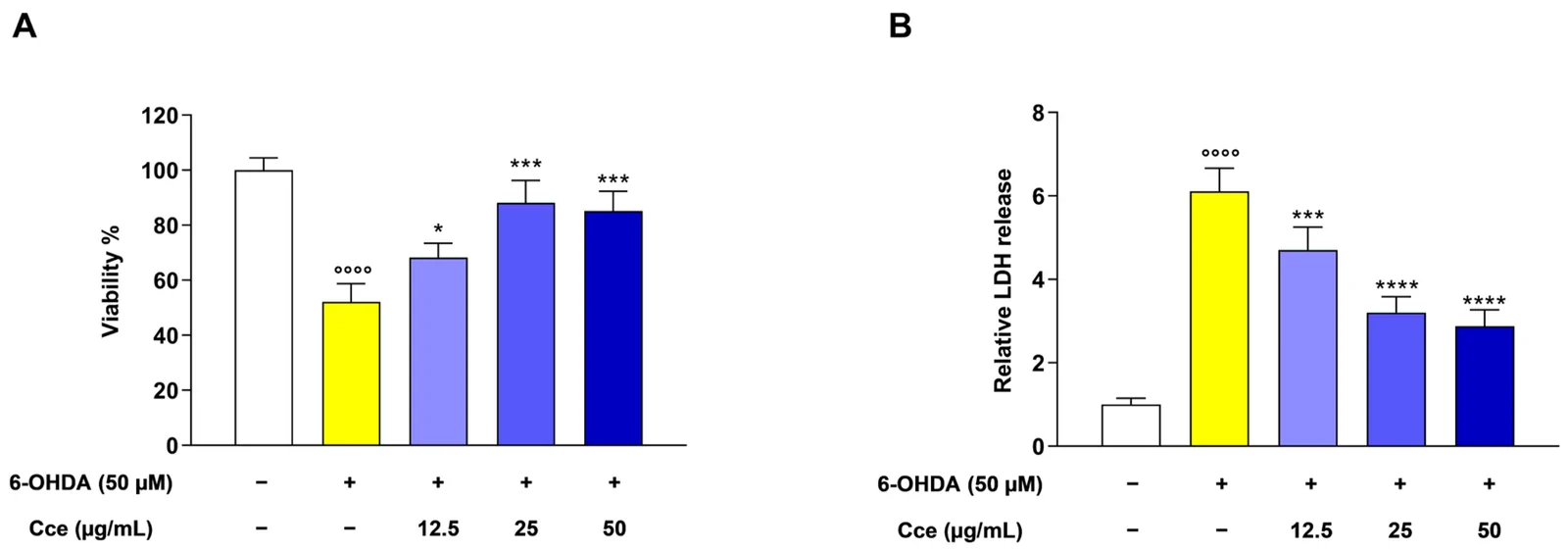
Protection of SH-SY5Y cell viability by Cce after 6-OHDA exposure. After pre-treatment with Cce (12.5, 25 and 50 µg/mL) of 1 h, 6-OHDA (50 µM) was added to cells for a further 24 h. (**A**) Cell viability was assessed by resazurin assay. Results are expressed as the percentage of fluorescence detected in cells compared to untreated ones, set as 100% (CTRL). (**B**) LDH levels are shown as values detected in cells relative to control ones (CTRL), which were arbitrarily expressed as 1. Data are expressed as means ± SEM of three independent experiments performed in eight replicates (*n* = 3). °°°° *p* < 0.0001 vs. CTRL; * *p* < 0.05, *** *p* < 0.001, and **** *p* < 0.0001 vs. 6-OHDA-exposed cells.

The exposure of SH-SY5Y cells to 6-OHDA for 24 h significantly decreased cell viability to 52.1 ± 6.6%, as assessed via the resazurin assay. The pre-treatment with Cce significantly hindered the effect of 6-OHDA. Indeed, the 12.5 µg/mL brought the viability to 68.2 ± 5.2%, while the 25 and 50 µg/mL to 88.1 ± 8.1% and 85.1 ± 7.2%, respectively (Figure 2A).

Moreover, 6-OHDA induced a significant release of LDH in the medium of cells up to 6.11 ± 0.55-fold compared with control cells, indicating an increase in cell death. Of note, the pre-treatment with 12.5 µg/mL lowered the LDH release to 4.7 ± 0.5-fold, while the 25 and 50 µg/mL to 3.2 ± 0.4 and 2.8 ± 0.4-fold, respectively (Figure 2B).

### 3.4. Cce Blocked Cell Cycle Progression in G2/M Phase of SH-SY5Y Cells Exposed to 6-OHDA

**Figure 3:**
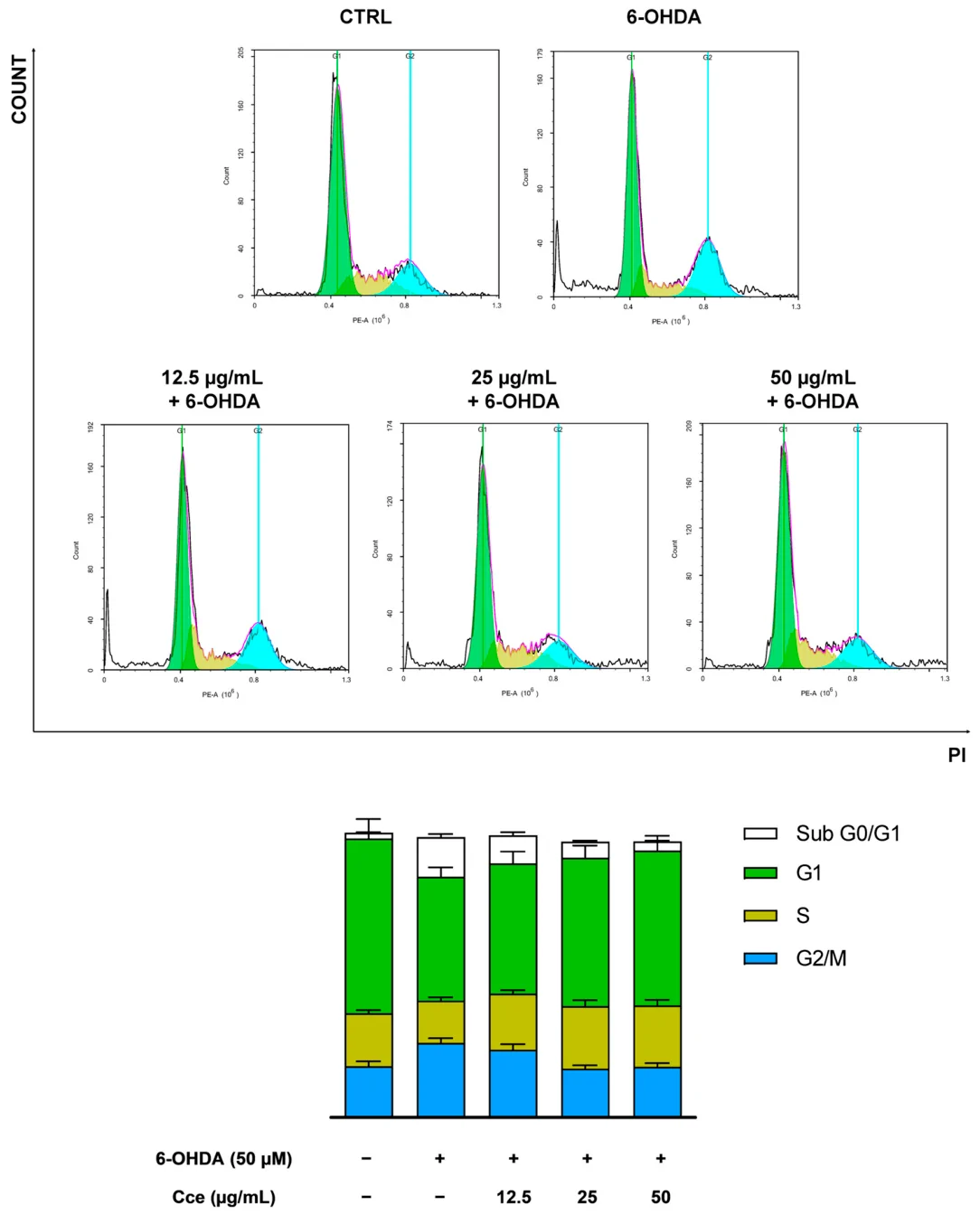
Effect of Cce on SH-SY5Y cell cycle progression after exposure to 6-OHDA. The pre-treatment with Cce (12.5, 25 and 50 µg/mL) lasted 1 h. Afterwards, 6-OHDA (50 µM) was added to cells for a further 24 h. The plots are representative of three different sessions performed in triplicate (*n* = 3). Percentages of cells populating each phase of the cell cycle are reported in the histograms ± SEM. Sub-G0/G1 is depicted in white, G1 in green, S in yellow and G2/M in cyan.

Ascertained the effect of 6-OHDA on the viability of SH-SY5Y cells, cell cycle distribution was investigated to appreciate whether this was influenced. The exposure of SH-SY5Y cells to 6-OHDA brought an increase in cells populating the G2/M phase (25.95 ± 1.7%, *p* < 0.05 vs. CTRL) compared with control cells (17.86 ± 1.7%), thus indicating a block of cell cycle in this phase. Of note, Cce pre-treatment was able to hinder this effect. Indeed, the 12.5, 25 and 50 µg/mL lowered the percentage of cells populating this phase to 23.64 ± 1.9%, 17.03 ± 1.15% (*p* < 0.01 vs. 6-OHDA) and 17.66 ± 1.25% (*p* < 0.01 vs. 6-OHDA), respectively (Figure 3).

Interestingly, 6-OHDA also increased cells in the sub-G0/G1 phase (13.81 ± 1.11%, *p* < 0.0001 vs. CTRL), typical of hypodiploid ones and hence undergoing apoptosis. As for the other phase, Cce was able to decrease the number of cells populating the sub-G0/G1 one. Indeed, the 12.5, 25 and 50 µg/mL lowered cells populating this phase to 9.81 ± 1.1% (*p* < 0.05 vs. 6-OHDA), 5.54 ± 0.44% (*p* < 0.0001 vs. 6-OHDA) and 3.19 ± 3.32% (*p* < 0.0001 vs. 6-OHDA), respectively (Figure 3).

### 3.5. Cce Hampered the Apoptosis Induced by 6-OHDA in SH-SY5Y Cells

**Figure 4:**
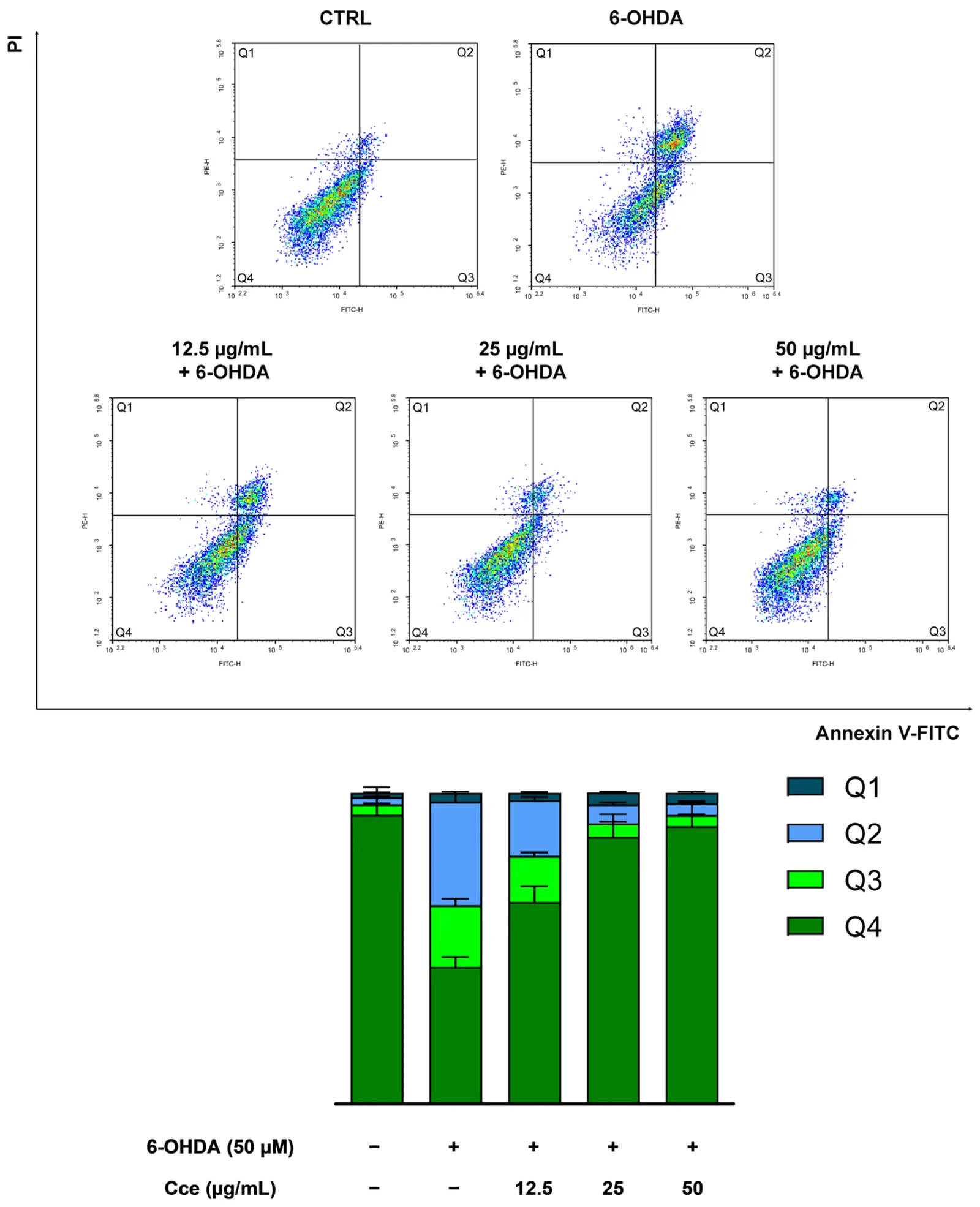
Assessment of apoptosis in SH-SY5Y cells pre-treated with Cce and exposed to 6-OHDA. The pre-treatment with Cce (12.5, 25 and 50 µg/mL) lasted 1 h. Afterwards, 6-OHDA (50 µM) was added to cells for a further 24 h. The detection of apoptosis was achieved by annexin V-FITC/PI staining. Representative annexin V/PI dot plots of three different experiments are presented. The quadrant named Q4 comprises viable cells, Q3 comprises cells in early apoptosis, Q2 comprises cells in late apoptosis, while Q1 comprises necrotic ones. Histograms represent the percentages of cell population present in the relative quadrants ± SEM of three experiments performed separately in triplicate (*n* = 3).

To explore the type of cell death induced by 6-OHDA in our cellular model and confirm the observations obtained during cell cycle analysis, we investigated the involvement of apoptosis by flow cytometric analyses. The stressor induced an increase in cells undergoing both early (19.81 ± 2.1%, *p* < 0.0001 vs. CTRL) and late (33.32 ± 2.4%, *p* < 0.0001 vs. CTRL) apoptosis. Interestingly, these effects were attenuated by pre-treatment of SH-SY5Y cells with Cce. Indeed, the 12.5 µg/mL was able to bring cells in both early and late apoptosis to 14.82 ± 1.1% (*p* < 0.001 vs. 6-OHDA) and 17.88 ± 1.1% (*p* < 0.0001 vs. 6-OHDA), respectively. The higher concentrations, namely 25 and 50 µg/mL of Cce, were able to lower cells populating early apoptosis to 4.32 ± 0.55 (*p* < 0.0001 vs. 6-OHDA) and 3.63 ± 0.23 (*p* < 0.0001 vs. 6-OHDA), respectively, and late one to 6.12 ± 0.58 (*p* < 0.0001 vs. 6-OHDA) and 3.73 ± 0.23 (*p* < 0.0001 vs. 6-OHDA), respectively (Figure 4).

### 3.6. Cce Modulated Gene Expression of Apoptotic-Related Markers

**Figure 5:**
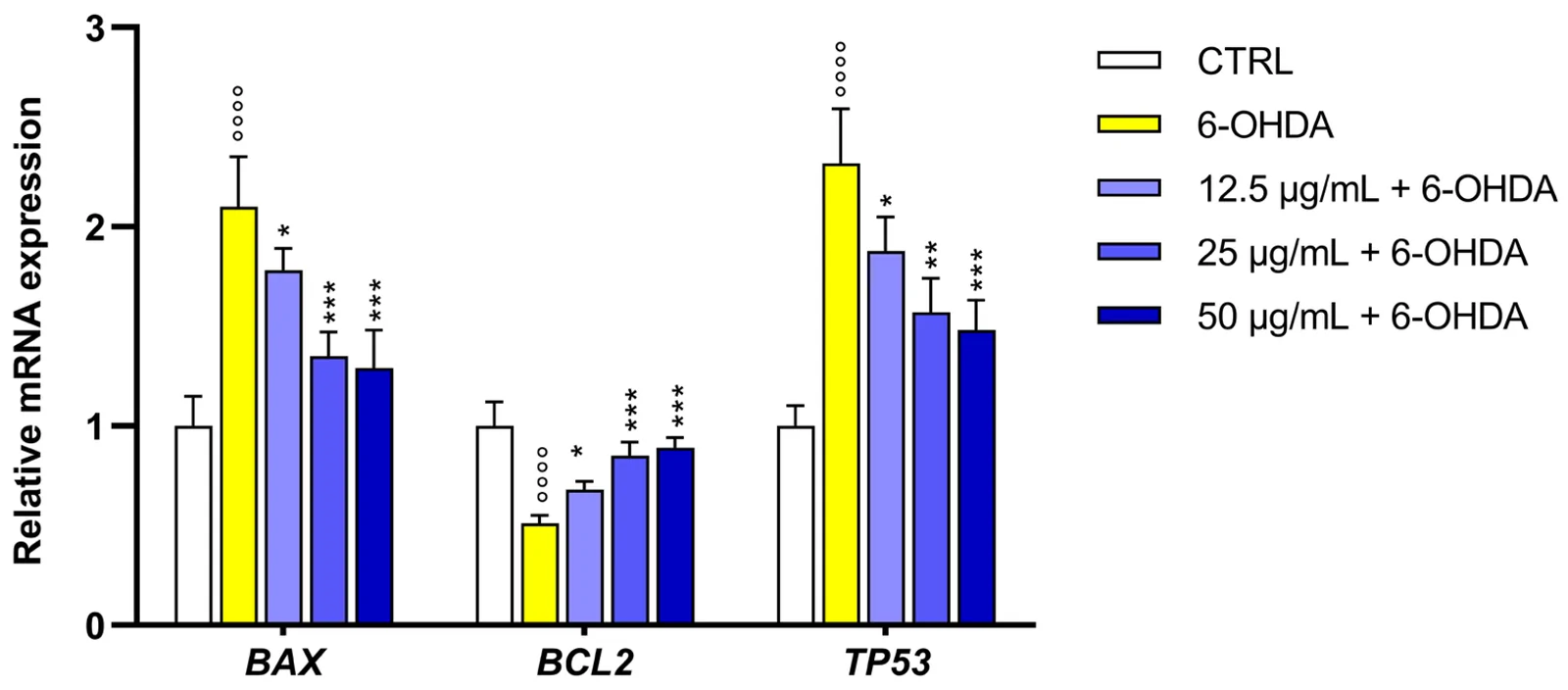
Effect of Cce on gene expression of apoptotic-related markers in SH-SY5Y cells pre-treated with Cce and exposed to 6-OHDA for 24 h. Relative quantities of mRNA, acquired by real-time PCR made in triplicate, were calculated by the 2^−∆∆Ct^ method, with β-actin (*ACTB*) as housekeeping gene. Results are expressed as fold change compared to untreated cells and expressed as mean ± SEM of three different experiments performed in triplicate (*n* = 3). °°°° *p* < 0.0001 vs. CTRL; * *p* < 0.05, ** *p* < 0.01, and *** *p* < 0.001 vs. 6-OHDA-exposed cells.

In order to better understand the molecular events sustaining the anti-apoptotic effects of Cce in 6-OHDA-stressed SH-SY5Y cells, we assessed the gene expression of the pivotal players in apoptotic machinery. Indeed, 6-OHDA increased levels of pro-apoptotic *BAX* and *TP53* by 2.1 ± 0.25-fold and 2.32 ± 0.27-fold, respectively, compared to control cells, whereas it decreased the level of the anti-apoptotic *BCL2* to 0.51 ± 0.04-fold. Interestingly, pre-treatment with Cce was able to revert this trend. Indeed, 12.5 µg/mL reduced gene expression of *BAX* and *TP53* to 1.78 ± 0.11 and 1.88 ± 0.17-fold, respectively, while increasing that of *BCL2* to 0.68 ± 0.04-fold. The 25 µg/mL reduced gene expression of *BAX* and *TP53* to 1.35 ± 0.12 and 1.57 ± 0.17-fold, respectively, while increasing that of *BCL2* to 0.85 ± 0.07-fold. Finally, the 50 µg/mL reduced gene expression of *BAX* and *TP53* to 1.29 ± 0.19 and 1.48 ± 0.15-fold, respectively, while increasing that of *BCL2* to 0.89 ± 0.05-fold (Figure 5).

### 3.7. Cce Reduced ROS Production in SH-SY5Y Cells Exposed to 6-OHDA

**Figure 6:**
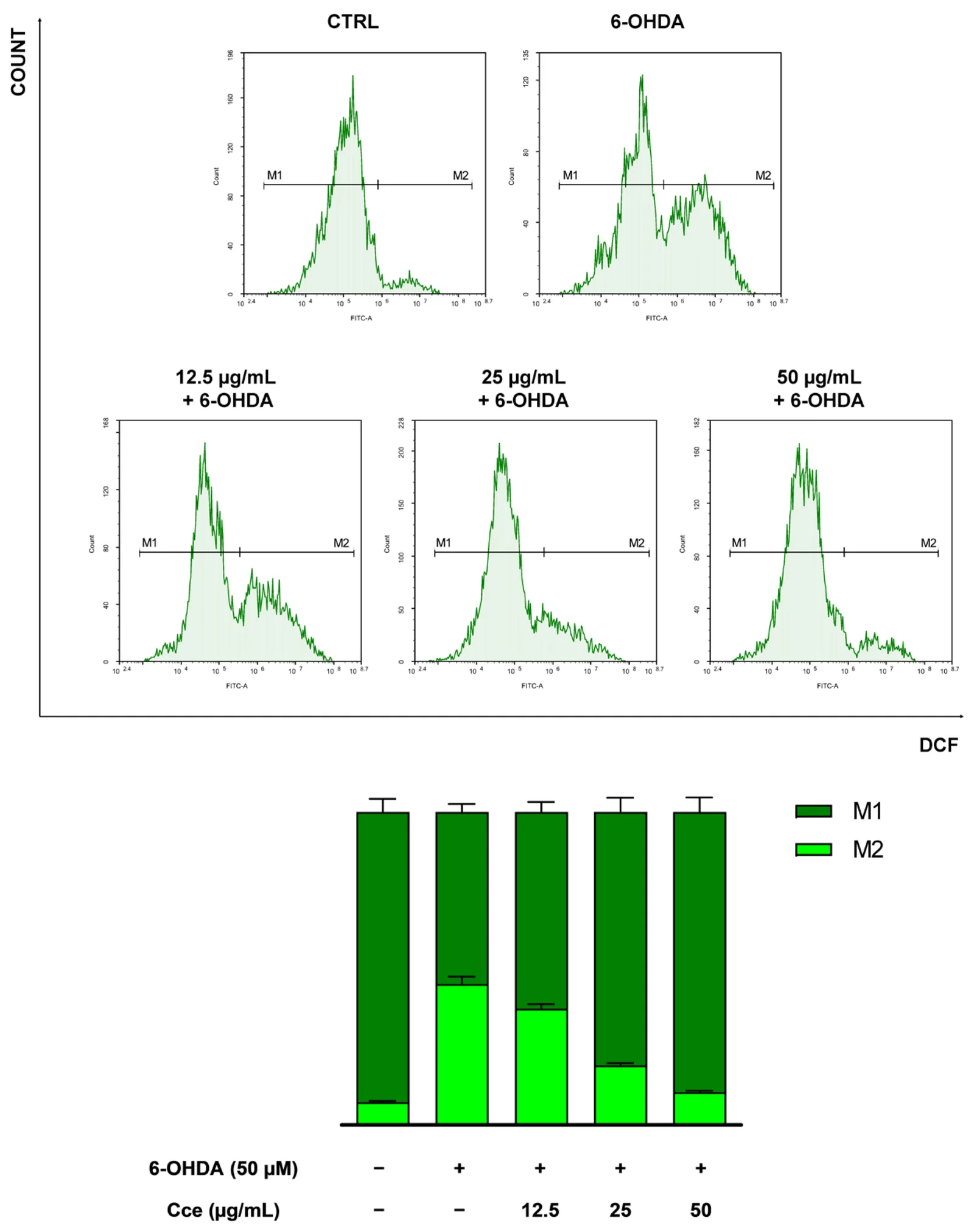
Cytofluorimetric assessment of ROS levels in SH-SY5Y cells pre-treated with Cce (12.5, 25 and 50 µg/mL) for 1 h and exposed to 6-OHDA (50 µM) for a further 6 h. ROS levels were quantified in terms of fluorescence exhibited by DCF by means of flow cytometry. The plots are representative of three different sessions, while the histograms represent the percentage ± SEM of non-fluorescent (M1) and fluorescent (M2) cells of three independent experiments performed in triplicate (*n* = 3).

Given the pro-oxidant effects of 6-OHDA, we investigated whether Cce pre-treatment could hamper ROS generation in differentiated SH-SY5Y cells via flow cytometry. The exposure of cells to 6-OHDA for 6 h brought a sharp increase in cellular ROS levels up to 45.02 ± 4.12% (*p* < 0.0001 vs. CTRL). The pre-treatment with Cce was able to reduce the percentage of fluorescent cells, thus with increased ROS. In fact, the 12.5, 25 and 50 µg/mL concentrations lowered ROS levels to 37.18 ± 2.5% (*p* < 0.01 vs. 6-OHDA), 19.03 ± 1.2% (*p* < 0.0001 vs. 6-OHDA) and 10.49 ± 0.8 (*p* < 0.0001 vs. 6-OHDA), respectively (Figure 6).

### 3.8. Cce Restored the Depleted Antioxidant Capacity of SH-SY5Y Cells Hampered by 6-OHDA

**Figure 7:**
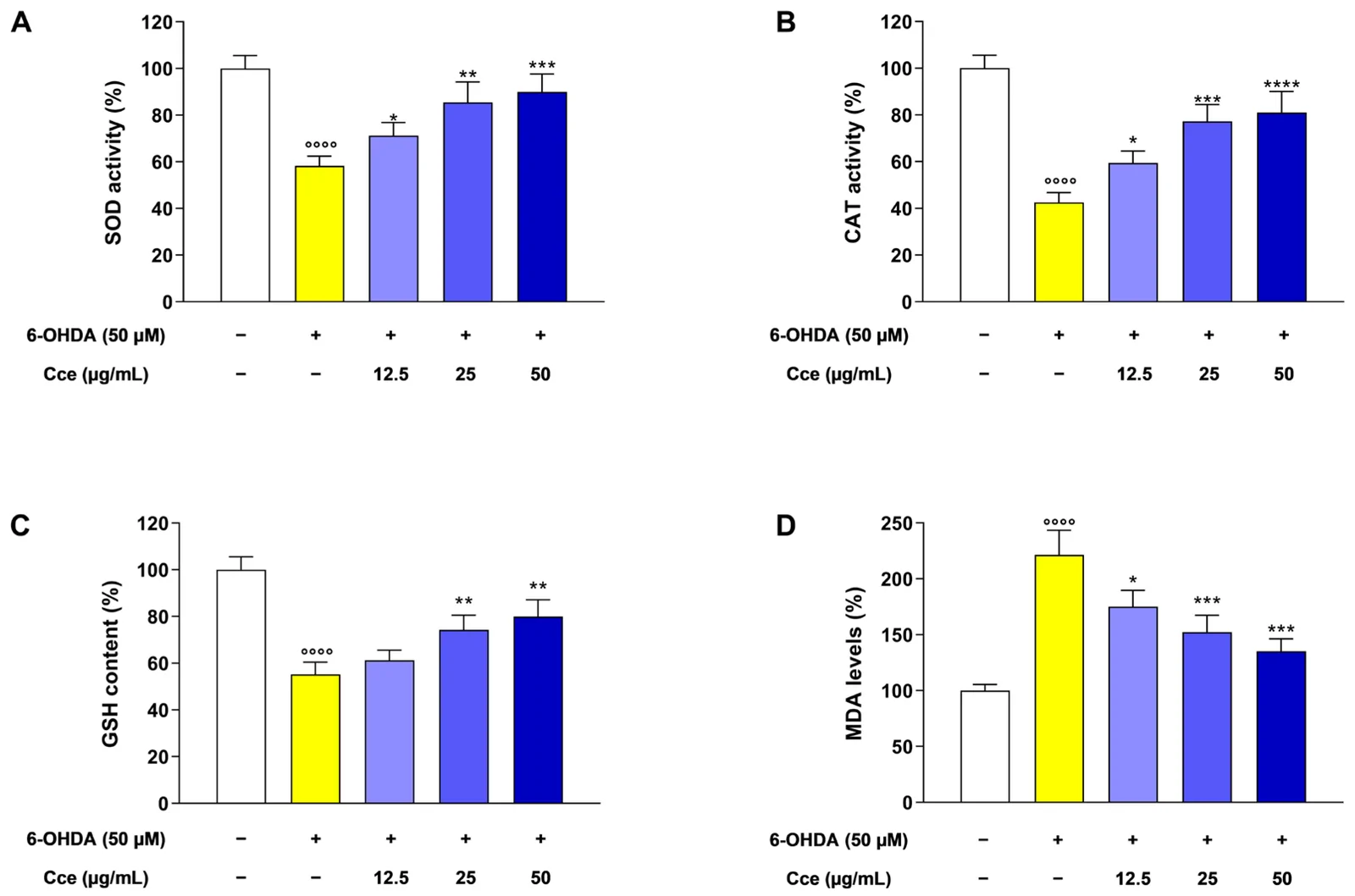
Antioxidant effects of pre-treatment with Cce for 1 h of SH-SY5Y cells exposed to 6-OHDA for 24 h. SOD (**A**) and CAT (**B**) activities, as well as GSH (**C**) and MDA (**D**) content, are expressed as the percentage compared to control cells and expressed as mean ± SEM of three different experiments performed in triplicate (*n* = 3). °°°° *p* < 0.0001 vs. CTRL; * *p* < 0.05, ** *p* < 0.01, *** *p* < 0.001 and **** *p* < 0.0001 vs. 6-OHDA-exposed cells.

To deeply investigate the antioxidant properties of Cce pre-treatment in SH-SY5Y cells exposed to 6-OHDA, we assessed the activity of both SOD and CAT enzymes. The stressor was able to significantly impede the activities of both SOD (58.24 ± 4.25%) and CAT (42.5 ± 4.25%) compared with control cells. Interestingly, we observed a restoration of their activities after Cce pre-treatment. Indeed, the 12.5, 25 and 50 µg/mL increased SOD activity up to 71.24 ± 5.65%, 85.4 ± 8.88% and 89.9 ± 7.77%, respectively, whereas CAT activity up to 59.45 ± 5.14%, 77.25 ± 7.15% and 81.1 ± 8.9%, respectively (Figure 7A,B).

The 6-OHDA exposure was also able to almost halve GSH reserve in SH-SY5Y cells (55.25 ± 5.24%) compared with control cells. For this parameter as well, Cce pre-treatment was able to restore the antioxidant capacity of stressed SH-SY5Y cells by bringing GSH levels to 61.25 ± 4.25%, 74.25 ± 6.25% and 79.95 ± 7.15% for the 12.5, 25 and 50 µg/mL concentrations, respectively (Figure 7C).

Moreover, this reflected also on the rate of lipid peroxidation quantified in terms of MDA levels. Indeed, 6-OHDA exposure increased MDA levels up to 221.25 ± 22.2% compared with control cells. The pre-treatment with 12.5, 25 and 50 µg/mL decreased MDA levels to 175.2 ± 14.5%, 152.2 ± 15.1% and 135.2 ± 11.2%, respectively (Figure 7D).

### 3.9. Cce Protected ΔΨm of SH-SY5Y Cells from the Oxidative Stress Induced by 6-OHDA

**Figure 8:**
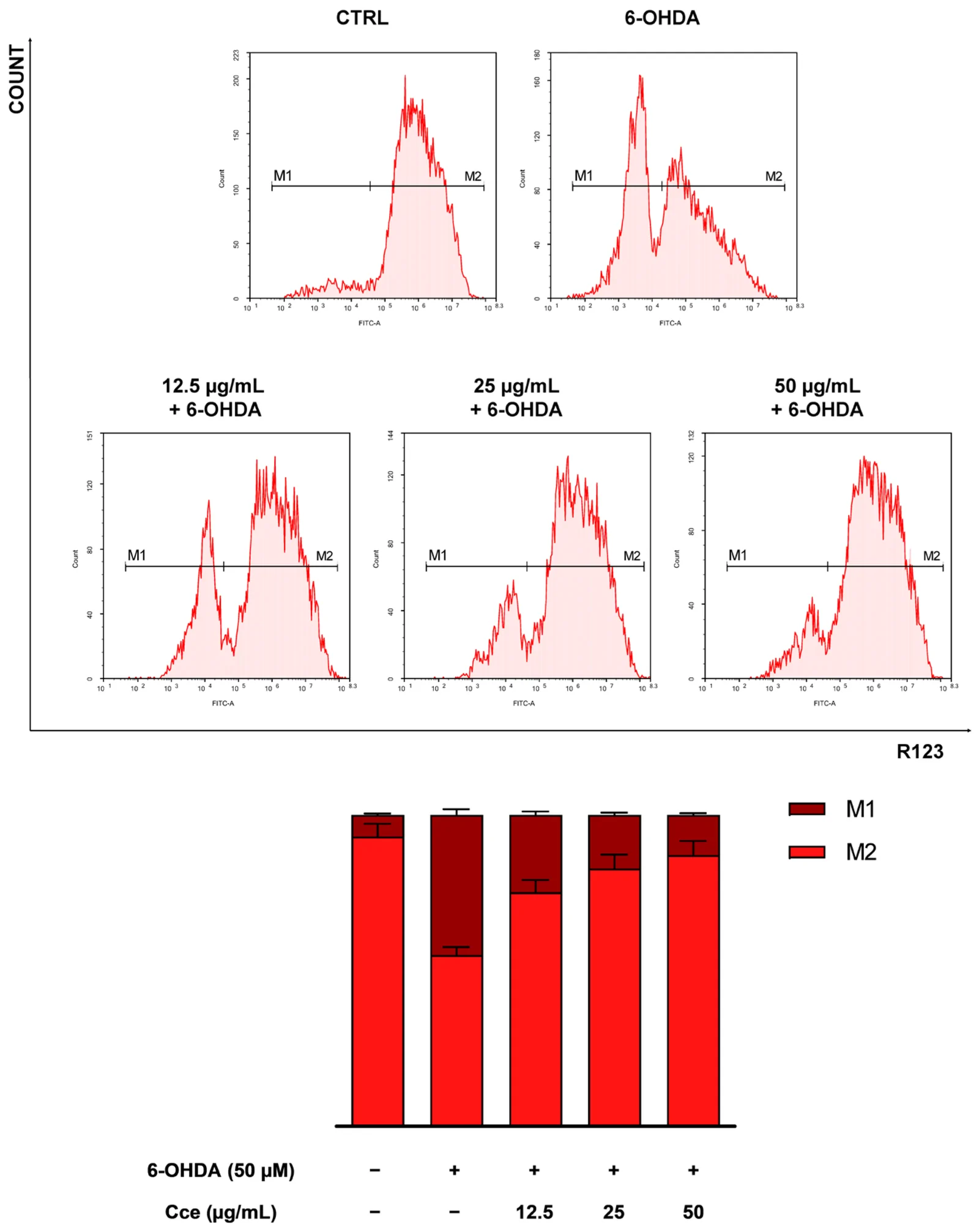
Evaluation of ΔΨm in SH-SY5Y cells pre-treated with Cce for 1 h and exposed to 6-OHDA for 6 h. The ΔΨm levels were quantified in terms of fluorescence exhibited by R123 by means of flow cytometry. The plots are representative of three different sessions, while histograms represent the percentage ± SEM of non-fluorescent (M1) and fluorescent (M2) cells of three independent experiments, performed in triplicate (*n* = 3).

Being mitochondria sensitive to pro-oxidant stimulus induced by 6-OHDA, we investigated the integrity of their membrane in terms of incorporation of R123 via flow cytometry. As expected, 6-OHDA caused damage to mitochondria in SH-SY5Y cells already after 6 h. Indeed, the number of fluorescent cells, thus with mitochondria able to retain the probe, drastically decreased (54.96 ± 4.44%, *p* < 0.001 vs. CTRL) respect control cells. Interestingly, Cce pre-treatment was able to revert this effect and increase the number of cells stained with R123 by 75.15 ± 6.8% (*p* < 0.05 vs. 6-OHDA), 82.73 ± 7.7% (*p* < 0.01 vs. 6-OHDA) and 87.03 ± 7.99% (*p* < 0.001 vs. 6-OHDA) for the 12.5, 25 and 50 µg/mL concentrations, hence suggesting a mitochondrial membrane potential restoration (Figure 8).

### 3.10. Cce Shielded Mitochondrial Integrity of SH-SY5Y Cells from 6-OHDA Exposure and Prevented Caspase Activation

**Figure 9:**
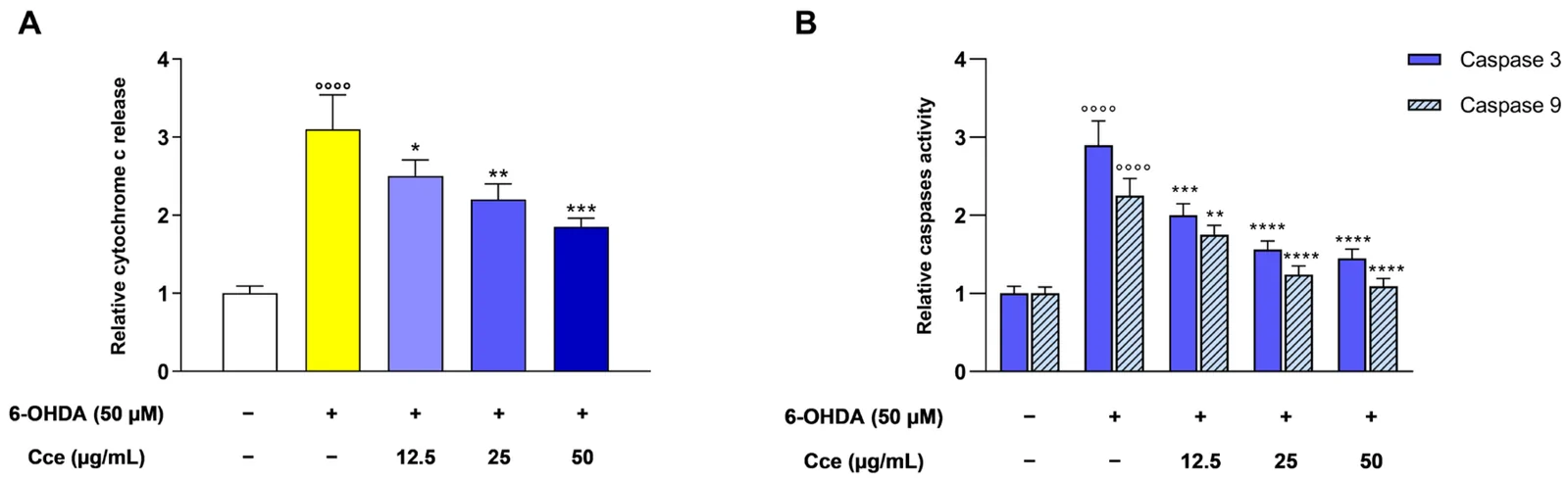
Release of cytochrome c (**A**) and effect on caspases 3 and 9 (**B**) in SH-SY5Y cells pre-treated with Cce for 1 h and exposed to 6-OHDA for a further 24 h. Results are expressed as relative to control cells, which was arbitrarily expressed as 1 and expressed as mean ± SEM of three different experiments performed in triplicate (*n* = 3). °°°° *p* < 0.0001 vs. CTRL; * *p* < 0.05, ** *p* < 0.01, *** *p* < 0.001 and **** *p* < 0.0001 vs. 6-OHDA-exposed cells.

Considering the loss of mitochondrial membrane potential caused by 6-OHDA, we investigated a marker of mitochondrial membrane disruption. Indeed, the stressor was able to significantly increase the release in the medium of SH-SY5Y cells of cytochrome c (3.1 ± 0.44-fold) compared to control cells. Notably, the pre-treatment with 12.5, 25 and 50 µg/mL of Cce decreased the release of cytochrome c to 2.5 ± 0.21, 2.2 ± 0.2 and 1.85 ± 0.1-fold, respectively (Figure 9A).

These effects were reflected in the activation of both caspase 3 and 9. Indeed, 6-OHDA increased the activation of both enzymes by 2.9 ± 0.31 and 2.25 ± 0.22-fold, clearly suggesting the initiation of the intrinsic apoptotic machinery. Interestingly, caspase 3 activation was brought to 2.0 ± 0.15, 1.56 ± 0.11 and 1.45 ± 0.12- fold after pre-treatment with 12.5, 25 and 50 µg/mL of Cce, respectively. In the same manner, caspase 9 activation was brought to 1.75 ± 0.12, 1.24 ± 0.11 and 1.09 ± 0.1-fold after pre-treatment with 12.5, 25 and 50 µg/mL of Cce, respectively (Figure 9B).

## 4. Discussion

Neurodegenerative diseases represent one of the major challenges facing modern medicine, and PD is among the most widespread worldwide [36]. In recent years, the scientific community has shown rising interest in plant-derived compounds, particularly polyphenols, for their health-promoting properties [37,38,39], among which neuroprotective one [40,41]. Anthocyanins, as a subclass of flavonoids, are recognised for their protective potential in neurodegenerative diseases [42,43]. This interest is grounded in an increasingly deeper understanding of the pathogenic mechanisms of PD, in which oxidative stress and mitochondrial dysfunction play a central and often initial role in the cascade of events leading to the death of dopaminergic neurons in the substantia nigra [44]. In support of this, growing experimental evidence demonstrates that polyphenolic compounds are capable of acting precisely on these molecular targets, exerting antioxidant activity, modulating mitochondrial function and activating cytoprotective signalling pathways, thus emerging as promising candidates for neuroprotective strategies in PD [13]. In this study, we investigated the mechanisms underlying the neuroprotective potential of Cce, in an in vitro model of PD.

The phytochemical composition of the extract is characterised by four main anthocyanins, namely cyanidin-3,5-O-diglucoside, followed by peonidin-3,5-O-diglucoside, cyanidin-3-O-glucoside and a complex derivative identified as cyanidin-coumaroyl glucoside-pyruvic acid, as we have previously reported [26].

To explore the neuroprotective potential, we employed differentiated SH-SY5Y cells exposed to 6-OHDA, which is a neurotoxin selective for dopaminergic neurons in the substantia nigra pars compacta. Its ability to reproduce the key biochemical features of PD, including oxidative stress, mitochondrial dysfunction and neuronal death via apoptosis, is supported by robust evidence, establishing it as one of the most reliable and widely validated experimental systems for the study of this disease [45,46]. From a mechanistic point of view, oxidative stress induced by 6-OHDA arises from its auto-oxidation which generates ROS and cytotoxic quinones. These molecules disrupt cellular redox homeostasis and lead to lipid peroxidation of the outer mitochondrial membrane. This oxidative damage compromises the maintenance of the mitochondrial membrane potential, promoting the opening of the mitochondrial permeability transition pore and the consequent release of cytochrome c into the cytosol. Cytochrome c, by binding to apoptotic protease activating factor-1, promotes the formation of the apoptosome and the cascade activation of initiator and effector caspases, thereby triggering the intrinsic apoptotic pathway characteristic of neuronal death in this model [47,48].

Prior to investigating the neuroprotective potential of Cce in this model, we assessed the safety profile of our extract in differentiated SH-SY5Y cells alone, along with in normal PBMCs. Notably, the extract was not able to alter the viability of either differentiated SH-SY5Y cells or PBMCs. For the latter, previous reports have claimed that extracts of different parts of *C. citrinus* (i.e., leaves and flowers) showed no cytotoxic effect in PMBCs [49].

In differentiated SH-SY5Y cells exposed to 6-OHDA, we showed that the stressor led to a marked reduction in the viability of SH-SY5Y cells, coupled to an increase in their death, whilst pre-treatment with Cce significantly counteracted this cytotoxic effect, suggesting a neuroprotective effect. In agreement with these findings, a mulberry fruit extract, containing anthocyanins, reduced cell death in the same cell model, with neuroprotective effects also confirmed in primary midbrain cultures stressed with 6-OHDA, as well as in vivo in mice stressed with 1-methyl-4-phenyl-1,2,3,6-tetrahydropyridine (MPTP) [50]. Moreover, an anthocyanin-rich extract from *Zea mays* L. var. ceratina alleviated cell death in SH-SY5Y cells exposed to hydrogen peroxide [51].

To better elucidate the mechanism of cell death induced by 6-OHDA in differentiated SH-SY5Y cells, we investigated whether the cell cycle was influenced. Indeed, 6-OHDA blocked cell cycle in G2/M phase in our cell model as a response to the increasing damage brought by the stressor. Interestingly, Cce pre-treatment was able to revert this outcome, restoring the normal cell cycle population. Other reports have highlighted the capacity of polyphenols to protect neuronal cell cycle from pro-oxidant stress. Indeed, hesperidin has been shown to restore a hampered cell cycle in our cell model [52].

Cell cycle analyses also shed light on the effect of 6-OHDA to increase cell population in sub-G0/G1 phase, characteristic of hypodiploid cells exiting the cell cycle and hence undergoing apoptosis. For this reason, we further investigated this mechanism employing flow cytometric analysis using Annexin V/PI staining. This technique allowed us to observe that treatment with 6-OHDA induced a significant increase in SH-SY5Y cells undergoing both early and late apoptosis, whereas pre-treatment with Cce was able to prevent apoptotic cell death and restored levels to those of the control cells. Furthermore, flow cytometric data were further confirmed at the transcriptional level by gene expression studies, which showed that 6-OHDA induced a significant overexpression of the pro-apoptotic *BAX* gene and a concomitant reduction in the expression of the anti-apoptotic *BCL2* gene, resulting in an altered *BCL2*/*BAX* ratio, effects coupled to an increase in the expression of *TP53*. Also in this case, Cce pre-treatment was able to significantly modulate gene expression of apoptotic markers to levels of control cells. The findings of this study are consistent with our previous studies conducted using the same cell model. Indeed, extracts of bergamot and mandarin juices reduced early and late apoptosis induced by 6-OHDA, with a concomitant reduction in the levels of *BAX* and *TP53*, along with restoration of *BCL2* expression [27,28]. Furthermore, in line with these data, cyanidin-3-O-glucoside has recently demonstrated similar anti-apoptotic properties in SH-SY5Y cells exposed to Aβ_1–42_, a model of Alzheimer’s disease [53], further supporting the neuroprotective role of anthocyanins in neurodegenerative diseases.

The pro-oxidant effect of 6-OHDA in differentiated SH-SY5Y cells is a well-known mechanism of the stress induced in this cell model [45]. For this reason, we investigated whether 6-OHDA-induced cell death was attributable to oxidative stress. Exposure to 6-OHDA induced a significant increase in ROS levels, whilst pre-treatment with Cce reduced oxidative status to parameters of control cells. Evidence of other anthocyanin-rich extracts able to reduce the pro-oxidant status of neurotoxins is robust. Indeed, myrtle berry by-product extracts and their procyanidin lowered the ROS production induced by 6-OHDA in PC12 cells [54], as well as in rats [55].

The ability of Cce to attenuate ROS in SH-SY5Y cells treated with 6-OHDA may be attributable to a dual mechanism. On the one hand, the anthocyanins in the extract possess documented radical scavenging activity. Indeed, we have previously demonstrated that the Cce actively neutralised DPPH, AAPH and ABTS radicals [16]. On the other hand, the reduction in cellular ROS appears to be mediated by the reactivation of endogenous antioxidant systems. Indeed, we observed that 6-OHDA significantly reduces the activity of SOD and CAT, the cellular main antioxidant enzymes, as well as reserve of GSH and expression of glutathione peroxidase activity. Of note, pre-treatment with Cce restored the activity of these enzymes, as well as GSH levels almost to baseline values. This protective effect is consistent with what has been documented for other polyphenolic compounds in cellular models of PD. Kesh and colleagues demonstrated that naringenin reduces 6-OHDA-induced levels of CAT, SOD and GSH in SH-SY5Y cells [56]. Similarly, Chen and colleagues reported that procyanidins restored the enzymatic activity of SOD and CAT reduced by 1-methyl-4-phenylpyridinium (MPP^+^) in PC12 cells, a well-known PD model, suggesting a mechanism comparable to that we hypothesise for the anthocyanins in Cce [57].

In parallel with the loss of the antioxidant protective system, we observed increased MDA levels in SH-SY5Y cells exposed to 6-OHDA. Indeed, it has been demonstrated that high ROS levels target lipids, proteins and nucleic acids, increasing MDA levels. This oxidation byproduct is able to impair mitochondrial integrity as well as its functionality, starting a vicious cycle and being one of the major contributors to ageing and neurodegenerative diseases [58]. This prompted us to investigate whether mitochondrial integrity was hampered in our cellular model. Indeed, 6-OHDA was able to strongly alter ΔΨm, as demonstrated by lesser retention of R123 within mitochondria. These findings are consistent with the mechanism of action of 6-OHDA in the SH-SY5Y model, in which the neurotoxin generates ROS, inducing oxidative stress and resulting in mitochondrial depolarisation [59,60]. These effects were hampered by pre-treatment with Cce, suggesting a robust protection of mitochondria elicited by our extract. As for the other parameters, evidence supports our results since myrtle berry by-product extracts, rich in anthocyanins, showed decreased MDA levels and protected mitochondrial functionality in 6-OHDA-stressed PC12 cells [54].

In order to confirm whether the mitochondrial damage could sustain the apoptotic cell death observed, we investigated cytochrome c release. It has been suggested that 6-OHDA induced mitochondrial fragmentation, which is an early event prior to the collapse of the mitochondrial membrane potential and the consequent cytochrome c release in SH-SY5Y cells [61]. In our cellular model, we observed a relevant release of cytochrome c after 6-OHDA exposure, suggesting robust mitochondrial damage. Interestingly, Cce pre-treatment was able to counteract this effect. This is in line with a previous report in which levels of released cytochrome c were reduced by an anthocyanin-rich fraction of black rice bran extract in SK-N-SH cells exposed to amyloid β [62].

The release of cytochrome c after mitochondrial damage is one of the main cellular drivers of the intrinsic apoptotic pathway, consequently activating the caspase cascade [63]. In particular, the activation of caspase 9 is claimed to be another cellular marker of mitochondrial damage and hence neuronal cell death [64]. Indeed, we observed that 6-OHDA was able to unleash the activation of caspase 9 and the effector caspase 3 in our cellular model. These effects were strongly inhibited by Cce pre-treatment, suggesting this as a plausible mechanism underlying the anti-apoptotic potential of the extract in neuronal cells. Other anthocyanin-rich extracts have been shown to inhibit caspase 9 and 3 activation in different models of neurodegeneration, hence supporting our results [62].

## 5. Conclusions

Overall, we showed that Cce hindered the effects of 6-OHDA in differentiated SH-SY5Y cells by protecting their viability and hindering their death. Moreover, Cce was able to prevent cell cycle dysregulation and reduce the number of cells undergoing apoptosis. In addition, Cce restored the antioxidant capacity along with reducing ROS levels in SH-SY5Y cells. This protected mitochondrial integrity, as appreciated by the preservation of membrane potential, thus leading to reduced cytochrome c release and activation of the intrinsic apoptotic pathway. Despite the limitations of an in vitro study, our results pave the way for further investigations in more complex experimental models to support the potential application of Cce in the management of neurodegenerative diseases, such as PD.

## Figures and Tables

**Table 1:** Oligonucleotide primer sequences employed for real-time PCR.

Gene Product	NCBI Reference Sequence	Primer Sequence
*BAX*	NM_138764.5	Forward: 5′-GGACGAACTGGACAGTAACATGG-3′ Reverse: 5′-GCAAAGTAGAAAAGGGCGACAAC-3′
*BCL2*	NM_000657.3	Forward: 5′-ATCGCCCTGTGGATGACTGAG-3′ Reverse: 5′-CAGCCAGGAGAAATCAAACAGAGG-3′
*TP53*	NM_000546.6	Forward: 5′-GTGTGGAGTATTTGGATGAC-3′ Reverse: 5′-ATGTAGTTGTAGTGGATGGT-3′
*ACTB*	NM_001101.5	Forward: 5′-TTGTTACAGGAAGTCCCTTGCC-3′ Reverse: 5′-ATGCTATCACCTCCCCTGTGTG-3′

## Data Availability

The original contributions presented in this study are included in the article. Further inquiries can be directed toward the corresponding author.

## Abbreviations

The following abbreviations are used in this manuscript:

6-OHDA	6-hydroxydopamine
CAT	Catalase
Cce	*Callistemon citrinus* anthocyanin-rich extract
CyG	Cyanidin 3 O glucoside
DCFH-DA	2′,7′-dichlorodihydrofluorescein
DE	Dried extract
FITC	Fluorescein-isothiocyanate
GSH	Glutathione
MDA	Malondialdehyde
PBMCs	Peripheral blood mononuclear cells
PI	Propidium iodide
ROS	Reactive oxygen species
RP HPLC DAD ESI MS/MS	Reverse phase high-performance liquid chromatography coupled to diode array detection and electrospray ionisation tandem mass spectrometry
SEM	Standard error of the means
SOD	Superoxide dismutase
